# Supplementary material for: Hot spot prediction in protein-protein interactions by an ensemble system
Source: BMC Syst Biol. 2018 Dec 31;12(Suppl 9):132. doi: 10.1186/s12918-018-0665-8 (PMC6311905; doi:10.1186/s12918-018-0665-8)
Supplement: Supplementary file 2 — All relevant MATLAB code. A simple Matlab implement of our predictor. (ZIP 19,742 kb) [file 12918_2018_665_MOESM2_ESM.zip › Additional file 2/Read ASA/ASA website/ASEdb/3HHRA.htm]

 NetSurfP - Protein Surface Accessibility and Secondary Structure Predictions

  

|  |  |  |
| --- | --- | --- |
|  |  | NetSurfP - Protein Surface Accessibility and Secondary Structure Predictions  Technical University of Denmark |

  


---

```
# For publication of results, please cite:
# A generic method for assignment of reliability scores applied to solvent accessibility predictions.
# Bent Petersen, Thomas Nordahl Petersen, Pernille Andersen, Morten Nielsen and Claus Lundegaard
# BMC Structural Biology 2009, 9:51 doi:10.1186/1472-6807-9-51
#
# Column 1: Class assignment - B for buried or E for Exposed - Threshold: 25% exposure, but not based on RSA
# Column 2: Amino acid
# Column 3: Sequence name
# Column 4: Amino acid number
# Column 5: Relative Surface Accessibility - RSA
# Column 6: Absolute Surface Accessibility
# Column 7: Z-fit score for RSA prediction
# Column 8: Probability for Alpha-Helix
# Column 9: Probability for Beta-strand
# Column 10: Probability for Coil
E F  3HHRA                  1    0.725 145.528  -1.608   0.003   0.003   0.994
E P  3HHRA                  2    0.581  82.472  -1.035   0.018   0.019   0.964
E T  3HHRA                  3    0.595  82.471  -0.125   0.058   0.017   0.925
E I  3HHRA                  4    0.342  63.233  -0.593   0.115   0.016   0.868
E P  3HHRA                  5    0.330  46.813   0.228   0.181   0.016   0.803
B L  3HHRA                  6    0.119  21.734  -0.372   0.782   0.003   0.216
E S  3HHRA                  7    0.499  58.483   1.008   0.923   0.002   0.076
E R  3HHRA                  8    0.471 107.836   0.642   0.923   0.002   0.076
B L  3HHRA                  9    0.053   9.778   0.974   0.970   0.001   0.030
B F  3HHRA                 10    0.073  14.651   0.468   0.970   0.001   0.030
E D  3HHRA                 11    0.396  57.020   0.184   0.970   0.001   0.030
B N  3HHRA                 12    0.270  39.543   0.841   0.970   0.001   0.030
B A  3HHRA                 13    0.024   2.667   0.384   0.970   0.001   0.030
B M  3HHRA                 14    0.090  18.009  -0.208   0.970   0.001   0.030
E L  3HHRA                 15    0.313  57.347   0.393   0.970   0.001   0.030
B R  3HHRA                 16    0.117  26.862   0.618   0.970   0.001   0.030
B A  3HHRA                 17    0.024   2.612  -0.123   0.923   0.002   0.076
E H  3HHRA                 18    0.341  62.046   0.256   0.923   0.002   0.076
B R  3HHRA                 19    0.281  64.418   0.776   0.970   0.001   0.030
B L  3HHRA                 20    0.030   5.438   0.519   0.970   0.001   0.030
B H  3HHRA                 21    0.209  37.963   0.491   0.970   0.001   0.030
E Q  3HHRA                 22    0.437  78.012   1.684   0.970   0.001   0.030
B L  3HHRA                 23    0.103  18.896   1.286   0.970   0.001   0.030
B A  3HHRA                 24    0.026   2.887   0.056   0.970   0.001   0.030
E F  3HHRA                 25    0.359  71.991   0.909   0.988   0.000   0.012
E D  3HHRA                 26    0.411  59.240   1.119   0.988   0.000   0.012
B T  3HHRA                 27    0.033   4.605   0.374   0.970   0.001   0.030
B Y  3HHRA                 28    0.114  24.340   0.982   0.970   0.001   0.030
E Q  3HHRA                 29    0.434  77.495   1.873   0.970   0.001   0.030
B E  3HHRA                 30    0.257  44.950   0.977   0.970   0.001   0.030
B F  3HHRA                 31    0.033   6.683   0.385   0.970   0.001   0.030
B E  3HHRA                 32    0.157  27.445   0.255   0.923   0.002   0.076
E E  3HHRA                 33    0.604 105.589   0.609   0.858   0.002   0.139
E A  3HHRA                 34    0.452  49.843   0.584   0.694   0.003   0.303
B Y  3HHRA                 35    0.145  30.965   0.707   0.406   0.004   0.590
B I  3HHRA                 36    0.072  13.357  -1.350   0.181   0.016   0.803
E P  3HHRA                 37    0.332  47.125  -0.444   0.115   0.016   0.868
E K  3HHRA                 38    0.536 110.173  -1.289   0.115   0.016   0.868
E E  3HHRA                 39    0.541  94.478  -1.320   0.115   0.016   0.868
B Q  3HHRA                 40    0.271  48.401  -1.027   0.184   0.043   0.773
B K  3HHRA                 41    0.292  60.147  -0.959   0.191   0.086   0.723
B Y  3HHRA                 42    0.306  65.392  -1.188   0.191   0.086   0.723
E S  3HHRA                 43    0.326  38.195  -1.632   0.278   0.093   0.628
E F  3HHRA                 44    0.386  77.530  -1.216   0.278   0.093   0.628
B L  3HHRA                 45    0.302  55.223  -1.078   0.307   0.165   0.527
E Q  3HHRA                 46    0.448  79.995  -0.270   0.307   0.165   0.527
E N  3HHRA                 47    0.340  49.805  -0.918   0.191   0.086   0.723
E P  3HHRA                 48    0.479  68.027  -1.095   0.113   0.043   0.844
E Q  3HHRA                 49    0.368  65.671  -1.510   0.053   0.043   0.903
E T  3HHRA                 50    0.353  48.989  -1.209   0.018   0.047   0.935
E S  3HHRA                 51    0.398  46.692  -1.932   0.018   0.047   0.935
B L  3HHRA                 52    0.224  41.069  -0.540   0.018   0.088   0.893
B C  3HHRA                 53    0.038   5.265  -1.224   0.018   0.088   0.893
B F  3HHRA                 54    0.165  33.095   0.001   0.018   0.088   0.893
B S  3HHRA                 55    0.207  24.225  -1.643   0.018   0.088   0.893
E E  3HHRA                 56    0.473  82.598  -1.539   0.019   0.141   0.840
E S  3HHRA                 57    0.316  37.023  -1.687   0.019   0.141   0.840
B I  3HHRA                 58    0.158  29.304  -0.214   0.019   0.141   0.840
E P  3HHRA                 59    0.559  79.265  -0.575   0.004   0.085   0.910
B T  3HHRA                 60    0.226  31.416  -0.318   0.005   0.015   0.979
B P  3HHRA                 61    0.262  37.135  -1.024   0.018   0.019   0.964
E S  3HHRA                 62    0.660  77.399  -0.852   0.018   0.019   0.964
E N  3HHRA                 63    0.557  81.516  -0.628   0.109   0.005   0.886
E R  3HHRA                 64    0.450 103.073  -0.341   0.406   0.004   0.590
E E  3HHRA                 65    0.637 111.197  -0.505   0.522   0.016   0.462
E E  3HHRA                 66    0.491  85.848   0.670   0.561   0.047   0.393
B T  3HHRA                 67    0.146  20.195  -0.751   0.502   0.102   0.396
E Q  3HHRA                 68    0.369  65.850   0.318   0.386   0.097   0.517
E Q  3HHRA                 69    0.720 128.592   0.383   0.199   0.152   0.649
B K  3HHRA                 70    0.197  40.585   1.111   0.113   0.087   0.800
E S  3HHRA                 71    0.584  68.468   0.098   0.018   0.047   0.935
E N  3HHRA                 72    0.414  60.624  -0.341   0.782   0.003   0.216
E L  3HHRA                 73    0.479  87.742  -0.295   0.923   0.002   0.076
E E  3HHRA                 74    0.375  65.565   0.238   0.970   0.001   0.030
B L  3HHRA                 75    0.046   8.349   0.221   0.970   0.001   0.030
B L  3HHRA                 76    0.056  10.327   0.551   0.970   0.001   0.030
E R  3HHRA                 77    0.329  75.226   0.497   0.970   0.001   0.030
B I  3HHRA                 78    0.101  18.741  -0.073   0.970   0.001   0.030
B S  3HHRA                 79    0.034   4.008  -1.182   0.970   0.001   0.030
B L  3HHRA                 80    0.119  21.807  -0.649   0.970   0.001   0.030
B L  3HHRA                 81    0.230  42.076  -0.960   0.988   0.000   0.012
B L  3HHRA                 82    0.066  12.011   0.118   0.970   0.001   0.030
B I  3HHRA                 83    0.064  11.877  -0.685   0.923   0.002   0.076
E Q  3HHRA                 84    0.292  52.080  -0.655   0.858   0.002   0.139
B S  3HHRA                 85    0.040   4.723  -1.384   0.782   0.003   0.216
B W  3HHRA                 86    0.055  13.252  -0.459   0.600   0.003   0.397
E L  3HHRA                 87    0.257  47.112  -0.632   0.694   0.003   0.303
E E  3HHRA                 88    0.352  61.442   0.027   0.502   0.002   0.495
B P  3HHRA                 89    0.043   6.130  -0.905   0.694   0.003   0.303
B V  3HHRA                 90    0.062   9.591  -0.950   0.923   0.002   0.076
E Q  3HHRA                 91    0.408  72.887   0.753   0.923   0.002   0.076
B F  3HHRA                 92    0.232  46.502   0.387   0.923   0.002   0.076
B L  3HHRA                 93    0.026   4.816   0.720   0.938   0.007   0.055
B R  3HHRA                 94    0.231  52.899  -0.200   0.938   0.007   0.055
E S  3HHRA                 95    0.450  52.705  -0.188   0.802   0.014   0.185
B V  3HHRA                 96    0.206  31.662  -0.682   0.600   0.003   0.397
B F  3HHRA                 97    0.128  25.730  -1.031   0.406   0.004   0.590
E A  3HHRA                 98    0.426  46.912  -1.253   0.176   0.004   0.820
E N  3HHRA                 99    0.467  68.369  -1.595   0.115   0.016   0.868
E S  3HHRA                100    0.354  41.512  -1.808   0.058   0.017   0.925
E L  3HHRA                101    0.443  81.095  -2.152   0.115   0.016   0.868
E V  3HHRA                102    0.377  57.945  -2.126   0.115   0.016   0.868
B Y  3HHRA                103    0.218  46.608  -0.915   0.184   0.043   0.773
B G  3HHRA                104    0.245  19.258  -1.218   0.268   0.043   0.689
E A  3HHRA                105    0.358  39.452  -1.785   0.354   0.048   0.598
E S  3HHRA                106    0.327  38.301  -0.326   0.455   0.046   0.498
B D  3HHRA                107    0.185  26.615  -1.336   0.717   0.014   0.269
E S  3HHRA                108    0.483  56.619   0.946   0.879   0.010   0.111
E N  3HHRA                109    0.350  51.255   0.551   0.923   0.002   0.076
B V  3HHRA                110    0.036   5.518  -0.258   0.970   0.001   0.030
E Y  3HHRA                111    0.262  56.054   0.502   0.970   0.001   0.030
E D  3HHRA                112    0.586  84.457   1.543   0.970   0.001   0.030
B L  3HHRA                113    0.190  34.862   1.054   0.970   0.001   0.030
B L  3HHRA                114    0.035   6.427  -0.086   0.970   0.001   0.030
E K  3HHRA                115    0.479  98.489   1.831   0.970   0.001   0.030
E D  3HHRA                116    0.362  52.222   2.189   0.970   0.001   0.030
B L  3HHRA                117    0.023   4.211   1.149   0.988   0.000   0.012
E E  3HHRA                118    0.278  48.567   0.918   0.988   0.000   0.012
E E  3HHRA                119    0.560  97.832   1.488   0.988   0.000   0.012
B G  3HHRA                120    0.138  10.821   0.296   0.988   0.000   0.012
B I  3HHRA                121    0.028   5.143   0.483   0.988   0.000   0.012
E Q  3HHRA                122    0.372  66.350   1.351   0.970   0.001   0.030
E T  3HHRA                123    0.285  39.460   1.384   0.970   0.001   0.030
B L  3HHRA                124    0.049   8.972   0.131   0.975   0.003   0.022
B M  3HHRA                125    0.064  12.846  -0.809   0.938   0.007   0.055
E G  3HHRA                126    0.519  40.814   0.551   0.879   0.010   0.111
E R  3HHRA                127    0.412  94.256   0.356   0.660   0.049   0.291
B L  3HHRA                128    0.089  16.387  -0.378   0.354   0.048   0.598
E E  3HHRA                129    0.338  58.961   0.000   0.113   0.087   0.800
E D  3HHRA                130    0.438  63.087  -1.350   0.113   0.087   0.800
E G  3HHRA                131    0.436  34.282  -1.500   0.053   0.043   0.903
B S  3HHRA                132    0.151  17.685  -1.963   0.053   0.043   0.903
E P  3HHRA                133    0.495  70.269  -0.892   0.113   0.087   0.800
E R  3HHRA                134    0.459 105.225  -1.340   0.113   0.087   0.800
E T  3HHRA                135    0.410  56.812  -1.627   0.113   0.087   0.800
E G  3HHRA                136    0.540  42.522  -2.061   0.118   0.150   0.732
E Q  3HHRA                137    0.495  88.336  -1.179   0.118   0.150   0.732
E I  3HHRA                138    0.300  55.426  -0.607   0.125   0.227   0.648
E F  3HHRA                139    0.305  61.314  -1.214   0.125   0.227   0.648
E K  3HHRA                140    0.383  78.845  -0.787   0.125   0.227   0.648
B Q  3HHRA                141    0.216  38.542  -0.456   0.064   0.216   0.721
E T  3HHRA                142    0.355  49.169  -1.695   0.056   0.142   0.802
B Y  3HHRA                143    0.278  59.344  -1.914   0.052   0.084   0.864
B S  3HHRA                144    0.200  23.417  -1.656   0.053   0.043   0.903
E K  3HHRA                145    0.625 128.624  -1.323   0.058   0.017   0.925
B F  3HHRA                146    0.259  52.021  -2.383   0.115   0.016   0.868
B D  3HHRA                147    0.227  32.754  -1.899   0.113   0.043   0.844
E T  3HHRA                148    0.402  55.716  -0.656   0.113   0.043   0.844
B N  3HHRA                149    0.295  43.261  -1.141   0.053   0.043   0.903
E S  3HHRA                150    0.439  51.427  -1.216   0.115   0.016   0.868
E H  3HHRA                151    0.585 106.484  -0.730   0.115   0.016   0.868
E N  3HHRA                152    0.510  74.679  -0.389   0.430   0.016   0.555
E D  3HHRA                153    0.387  55.723  -1.767   0.622   0.015   0.363
E D  3HHRA                154    0.458  65.969  -0.313   0.802   0.014   0.185
E A  3HHRA                155    0.407  44.895   0.563   0.802   0.014   0.185
B L  3HHRA                156    0.102  18.676   0.986   0.802   0.014   0.185
B L  3HHRA                157    0.205  37.462   0.202   0.802   0.014   0.185
E K  3HHRA                158    0.370  76.027  -0.177   0.717   0.014   0.269
B N  3HHRA                159    0.200  29.309  -0.468   0.622   0.015   0.363
B Y  3HHRA                160    0.156  33.423   0.769   0.430   0.016   0.555
E G  3HHRA                161    0.335  26.333  -1.003   0.430   0.016   0.555
B L  3HHRA                162    0.057  10.418   0.121   0.430   0.016   0.555
B L  3HHRA                163    0.077  14.099  -1.058   0.622   0.015   0.363
B Y  3HHRA                164    0.287  61.353   0.026   0.622   0.015   0.363
B C  3HHRA                165    0.050   7.062  -1.006   0.622   0.015   0.363
B F  3HHRA                166    0.072  14.531  -0.034   0.561   0.047   0.393
E R  3HHRA                167    0.344  78.753   0.713   0.354   0.048   0.598
E K  3HHRA                168    0.424  87.279   0.649   0.257   0.016   0.727
B D  3HHRA                169    0.153  22.105  -0.931   0.339   0.016   0.645
B M  3HHRA                170    0.107  21.491  -1.025   0.600   0.003   0.397
E D  3HHRA                171    0.470  67.741  -0.083   0.858   0.002   0.139
E K  3HHRA                172    0.246  50.643   1.638   0.923   0.002   0.076
B V  3HHRA                173    0.021   3.228   1.222   0.970   0.001   0.030
E E  3HHRA                174    0.326  56.970   0.496   0.970   0.001   0.030
B T  3HHRA                175    0.157  21.707   0.231   0.970   0.001   0.030
B F  3HHRA                176    0.064  12.785   1.030   0.970   0.001   0.030
B L  3HHRA                177    0.052   9.594   1.261   0.970   0.001   0.030
E R  3HHRA                178    0.399  91.394   1.520   0.938   0.007   0.055
B I  3HHRA                179    0.085  15.743   0.284   0.938   0.007   0.055
B V  3HHRA                180    0.062   9.576   0.269   0.802   0.014   0.185
E Q  3HHRA                181    0.330  58.956   0.815   0.605   0.105   0.290
B C  3HHRA                182    0.064   8.943  -0.282   0.453   0.248   0.299
E R  3HHRA                183    0.337  77.173   0.491   0.252   0.423   0.325
B S  3HHRA                184    0.256  30.003  -0.279   0.148   0.418   0.435
B V  3HHRA                185    0.217  33.338   0.710   0.118   0.150   0.732
E E  3HHRA                186    0.598 104.540   0.447   0.113   0.043   0.844
E G  3HHRA                187    0.659  51.848  -0.097   0.115   0.016   0.868
E S  3HHRA                188    0.504  59.034  -0.729   0.115   0.016   0.868
B C  3HHRA                189    0.235  33.008  -1.853   0.058   0.017   0.925
E G  3HHRA                190    0.800  62.984  -0.279   0.003   0.003   0.994

Explain the output.  Go back.
```

---
